# Crystal Structure and Magnetic Properties of the Novel Compound ErMn_5_Ge_3_

**DOI:** 10.3390/ma18020359

**Published:** 2025-01-14

**Authors:** Nidong Yang, Yunxiang Yang, Hui Luo, Shuohai Fang, Tianhua Ju, Shengyuan Lei, Wei He

**Affiliations:** MOE Key Laboratory of New Processing Technology for Nonferrous Metals and Materials, School of Resources, Environment and Materials, Guangxi University, Nanning 530004, China; 15937625790@163.com (N.Y.); yang15978058832@163.com (Y.Y.); luohui961022@163.com (H.L.); fangshuohai@163.com (S.F.); jutianhua@gxu.edu.cn (T.J.)

**Keywords:** ErMn_5_Ge_3_, X-ray diffraction, crystal structure, magnetic property, first principles, electronic structure

## Abstract

The RE-M-Ge systems (RE: rare earths, M: transition group elements) contain a large number of compounds with special magnetic properties. A novel compound ErMn_5_Ge_3_ was found during the investigation on the phase diagram of the Er-Mn-Ge ternary system, and its crystal structure and magnetic properties were investigated. Powder X-ray diffraction results show that ErMn_5_Ge_3_ crystallizes in an orthorhombic YNi_5_Si_3_-type structure with the space group Pnma (No. 62) and the lattice parameters of a = 13.0524(6) Å, b = 3.8853(7) Å, and c = 11.4027(4) Å. The magnetization curves and isothermal magnetization curves from 100 to 300 K were measured for ErMn_5_Ge_3_. Magnetic tests showed that the compound was weakly magnetic and had a Curie temperature of 304 K. It is believed that its magnetic properties are determined by Mn atoms, which are surrounded by a complex environment, leading to uncertainty in the direction of the magnetic moment and hence poor magnetic ordering. This uncertainty simultaneously leads to a significant separation of the ZFC and FZ curves. First-principles calculations confirm that the magnetic properties of ErMn_5_Ge_3_ are mainly provided by the Mn atoms, and its magnetic moment is calculated to be about 4.5 μ_B_. A possible magnetic structure model with simultaneous Mn-Mn ferromagnetic/antiferromagnetic coupling is constructed based on the Mn atom spacing, which can well explain the magnetic performance of ErMn_5_Ge_3_.

## 1. Introduction

The RE-M-Ge systems (RE: rare earths, M: transition group elements) contain a large number of compounds with a variety of structures. Due to the complex interactions between the localized f-electrons of RE atoms and the delocalized d-electrons of M atoms, they usually exhibit complex electrical and magnetic properties [1]. Among them, the RE-Mn-Ge systems are generally framed by Mn-Ge polyhedra, and due to the rich and diverse interactions among RE, Mn, and Ge atoms, a number of materials with special magnetic properties have emerged in these systems.

The RE-Mn-Ge ternary compounds REMnGe, REMn_2_Ge_2_, and REMn_6_Ge_6_ have a variety of unique magnetic structures, and their magnetic properties have attracted much interest. Klosek et al. [2] studied the crystal and magnetic structure of the ErMnGe compound, which crystallizes in TiNiSi type (space group: Pnma) and exhibits antiferromagnetic ordering of the Mn and Er sublattice above room temperature. ErMnGe is a simple co-linear antiferromagnet at 2 K and the values of the magnetic moments are μ_Mn_ = 3.25(18) μ_B_ and μ_Er_ = 7.51(13) μ_B_ [2]. The Curie–Weiss temperature value is θp = −32(2) K, indicating the dominant role of the antiferromagnetic phase in ErMnGe. REMn_2_Ge_2_ has a unique layered structure, which is a natural superlattice structure [3]. A number of studies have demonstrated that many REMn_2_Ge_2_ (RE = Gd, Tb, and Dy) compounds exhibit distinctive magnetic phase transition behaviors, and their structural magnetic properties are mainly determined by the spacing of Mn atoms between and within the layers [3,4,5]. Leciejewicz et al. [6] carried out a neutron diffraction study of the magnetic ordering of ErMn_2_Ge_2_ and found ferromagnetic ordering at 4.2 K. It was further found that Er has a ferromagnetically ordered magnetic moment located at the basal plane, while Mn has an antiferromagnetically ordered magnetic moment parallel to the c-axis. REMn_6_Ge_6_ compounds consist of RE and Mn layers stacked alternately along the c-axis, and a giant magnetoresistance effect has been observed in some materials [7,8,9,10]. Uhlírová et al. [11] investigated the magnetization behavior of single-crystal ErMn_6_Ge_6_ from 2 to 600 K. This compound exhibits an antiferromagnetic structure at 475 K and undergoes a second magnetic ordering transition at 120 K.

It is noteworthy that the interactions between Mn atoms are mentioned in almost all of these compounds as having a significant influence on their magnetic properties. The Mn-Mn spacing can directly influence the form of magnetic coupling between Mn atoms, which generally exhibit antiferromagnetic coupling when the distances are short and prefer ferromagnetic coupling between Mn atoms when the distances are above a certain critical value [11,12,13,14]. This distance varies slightly with different crystal structures and temperatures. This phenomenon has been observed in many different Mn-containing materials [11,12,13,14], and the relationship between the spacing of Mn atoms and their magnetic coupling has also been used to explain the ferromagnetic/antiferromagnetic coupling process in some materials [15].

REM_5_Ge_3_ (M = Mn, Ni, Pd, Pt) compounds crystallized in the orthorhombic YNi_5_Si_3_-type structure with the space group Pnma (No. 62). Studies have shown that the rare-earth elements show a one-dimensional linear arrangement in the REM_5_Ge_3_ structure, but due to their far spacing, direct interaction cannot be realized in most cases. Only in some materials containing heavy elements with strong spin–orbit coupling (e.g., Pd or Pt), they can remain magnetically coupled and form the corresponding one-dimensional magnetic structure [16,17,18]. For the case of M = Mn, only the TbMn_5_Ge_3_ compound has been identified among the REM_5_Ge_3_ compounds. Magnetic tests of TbMn_5_Ge_3_ showed that Mn atoms exhibit both ferromagnetic and antiferromagnetic coupling, determining the overall magnetic properties [19].

During the investigation on the phase diagram of the Er-Mn-Ge ternary compounds, we have discovered a new compound, ErMn_5_Ge_3_. The purpose of this paper is to investigate the structure and magnetic properties of the novel ErMn_5_Ge_3_ compound and try to explain its magnetic behavior.

## 2. Materials and Methods

Polycrystalline samples of ErMn_5_Ge_3_ were prepared by arc melting a stoichiometric amount of high-purity initial metals in a high-purity argon atmosphere. In order to compensate for the loss of Mn during the smelting process, an extra amount of 5 wt.% Mn was added. The sample was repeatedly melted 4 times to ensure uniformity, and the weight loss of the ingot was less than 1 wt.%. The sample was sealed in evacuated quartz tubes and annealed at 1073 K for 3 weeks for homogenization annealing, and finally quenched into an ice–water mixture. It has to be noted that to prepare a sample with a single phase of ErMn_5_Ge_3_ is very difficult. Even with the correct and accurate elemental ratio, samples containing only the ErMn_5_Ge_3_ phase without any impurity phase were not obtained. A lot of time was spent on exploring the ErMn_5_Ge_3_ annealing process, and finally several relatively pure samples with the ErMn_5_Ge_3_ phase were obtained from dozens of samples. The sample with the most purity of the ErMn_5_Ge_3_ phase was selected for subsequent testing. The powder X-ray diffraction (XRD) patterns of the selected ErMn_5_Ge_3_ sample show that it contained mainly ErMn_5_Ge_3_ with slight amounts of ErMn_2_Ge_2_ and Er_2_O_3_ as impurities.

An Anton Paar XRDynamic500 diffractometer with Cu Kα radiation (made by Anton Paar, located in Graz, Austria) was used to collect the powder XRD data for ErMn_5_Ge_3_ in step-scan mode with the step size of 0.01° and stayed 1 s at each step. The 2θ range was set to be 10–110°. The collected XRD data were used for phase analysis and Rietveld refinement of the sample.

The magnetization curve and hysteresis loop of the sample were measured by Lake Shore 7410 Vibrating Sample Magnetometer (VSM) (made by Lake Shore, located in Westerville, OH, USA) from 100 to 330 K with a maximum magnetic field of 15,000 Oe, and one piece with a mass of about 25 mg taken from the cast sample was used for magnetic testing. Afterwards, the magnetization–temperature curve was measured from 90 to 400 K in a field-cooled (FC) process and zero-field-cooled (ZFC) processes on the same instrument under an applied field of 1 kOe.

Finally, the generalized gradient approximation (GGA) exchange and related density functional theory (DFT) were utilized to calculate the energy, electronic structure, and magnetic coupling of ErMn_5_Ge_3_. All related calculations are completed through the Vienna ab initio simulation package (VASP 4.6) [20]. The projection augmented plane-wave (PAW) method [21] was used to establish a plane-wave basis set, calculate the total energy and force, and optimize the geometric structure, and perform DFT calculations using PW91 exchange and correlation functions. The atomic pseudopotentials used in the calculations are PAW_GGA, and the VRHFINs are Er: [core = Xe4], Mn: d6 s1, and Ge: s2p2. In performing the structural optimization, the cutoff energy were set to be 300, 350, and 400 eV for the trial, and the results show that stable convergence is already possible at 300 eV. In order to ensure the efficiency of the calculations, the energy cutoff was set to 300 eV, and the convergence values of energy and force were set to 10^−5^ eV and 0.01 eV/A, respectively. The Monkhorst-Pack scheme [22] was used to select the k-point grid, which is 16 × 4 × 4. Considering that Mn-Ge is a highly localized system with strong electron correlation (the 3D orbital electrons of the transition element Mn have a strong correlation in its compound system), during the calculation process, the GGA + U method [23] is used to accurately describe the electronic structure of Mn atoms by adding Coulomb potential *U* and exchange potential *J*, which reflect the strong correlation of 3D electrons. Even for the same element, there are still certain differences in the values of *U* and *J* in different compounds. Considering that Mn serves as the backbone of the structure and there are a large number of Mn-Mn interactions in the system, relatively large values are used here for both *U* and *J*. Here, we refer to various Mn-Ge systems to add parameters for the 3D orbitals of Mn atoms: *U* = 6.0 eV and *J* = 1 eV.

## 3. Results and Discussion

### 3.1. Crystal Structure

The XRD patterns of the ErMn_5_Ge_3_ samples were first analyzed using Jade 6.0 software [24]. The XRD patterns were successfully indexed in an orthorhombic structure and the lattice constants were found to be a = 13.0532(7) Å, b = 3.8854(2) Å, and c = 11.4026(8) Å. The reflection conditions (hkl: k + l = 2n; hk0: h = 2n; h00: h = 2n; 0k0: k = 2n; 00l: l = 2n) were pointed to two space groups: Pnma (No. 62) and Pna2_1_ (No. 33). Due to the higher symmetry of the space group of Pnma, it was first considered and chosen for the crystal structure of ErMn_5_Ge_3_. Based on the density measured by the specific gravity bottle method (D_m_ = 7.58 g/cm^3^) combined with the above obtained lattice parameters, it was found that the crystal structure of ErMn_5_Ge_3_ contained 18 atoms and two formulas (Z = 2) in each unit cell. Compared with the lattice parameters, XRD patterns, and measured density, the ErMn_5_Ge_3_ compound was found to be isostructural with TbMn_5_Ge_3_ [19]. Thus, the space group of the ErMn_5_Ge_3_ compound was further assigned to be Pmma (No. 62).

The lattice constants of the new compound ErMn_5_Ge_3_ obtained by the Jade6.0 program and the atomic parameters of TbMn_5_Ge_3_ were used as initial parameters to input into the GSAS-II program for the Rietveld refinement [25]. The crystallographic data of TbMn_5_Ge_3_ are available in the Inorganic Crystal Structure Database (ICSD) [19]. In the refinement, Er atoms adopt the Wyckoff siteof Tb atoms. The Pseudo-Voigt function was selected as the profile fitting function. In the Rietveld refinement, more than 20 parameters such as lattice parameters, FWHM parameters, peak shape parameters, preferred orientation parameters, atomic sites, and temperature factors were refined. The main results are given in Table 1.

**Figure 1:**
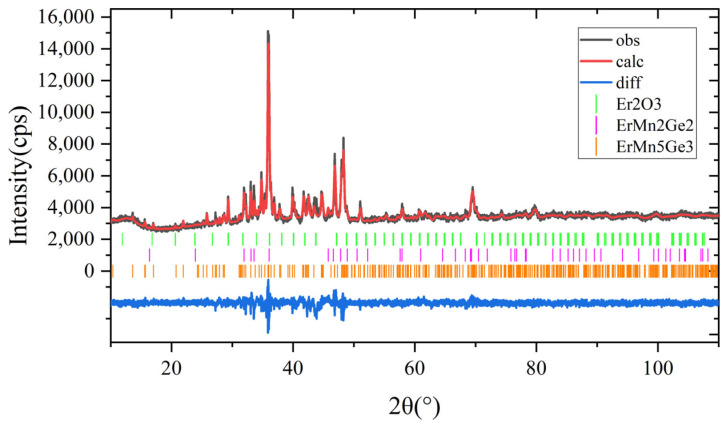
Powder XRD patterns of the new compound ErMn_5_Ge_3_.

Figure 1 presents the powder XRD patterns of ErMn_5_Ge_3_. The Rietveld refinement results further point out that ErMn_5_Ge_3_ is isostructural with TbMn_5_Ge_3_ and belongs to the YNi_5_Si_3_-type orthorhombic structure with the space group of Pnma (No. 62). Thus, the space group of Pnma (No. 62) but not Pna2_1_ (No. 33) is assigned to ErMn_5_Ge_3_. The refined lattice parameters for ErMn_5_Ge_3_ are obtained to be a = 13.0524(6) Å, b = 3.8853(7) Å, and c = 11.4027(4) Å. The atomic site of each atom of ErMn_5_Ge_3_ is given in Table 2. The refinement results also indicated that the sample mainly consists of 90.3 wt. % of ErMn_5_Ge_3_ with a small amount of ErMn_2_Ge_2_ (6.5 wt. %) and Er_2_O_3_(3.4 wt. %).

**Figure 2:**
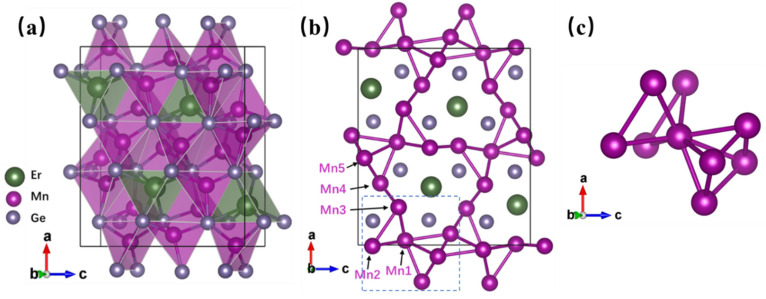
(**a**) The crystal structure of ErMn_5_Ge_3_. (**b**) Arrangement of Mn atoms in the structure, projected along [010]. (**c**) Mn atomic arrangements in the dashed part of (**b**).

**Figure 3:**
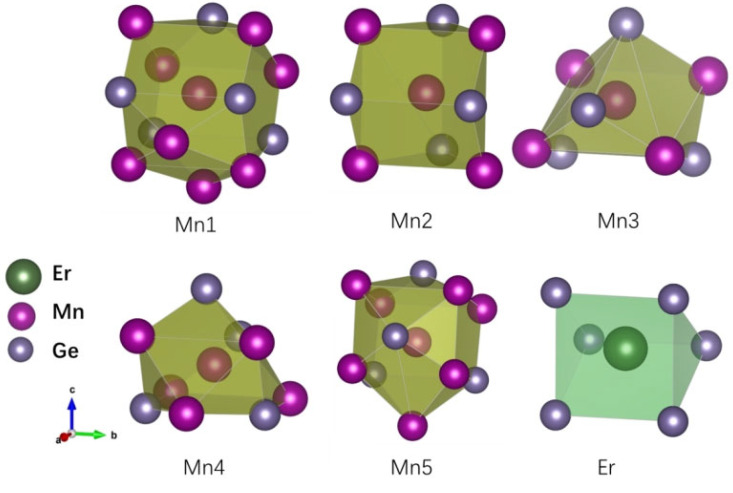
The environment of the nearest neighbor atoms of each Er and Mn atoms in the structure of the compound ErMn_5_Ge_3_.

The crystal structure of the new compound ErMn_5_Ge_3_ is shown in Figure 2a (VESTA software (vasp.5.4.1) [26] utilized). It can be seen that the polyhedra with Ge at the top corner forms the base structure of ErMn_5_Ge_3_, while the Mn and Er atoms are in the center of the polyhedra. The rare-earth atoms are linearly arranged along the [010] direction in the structure. There are five Wykoff sites of Mn atoms in the structure as shown in Figure 2b, and each of them stays in a different environment and is adjacent to 4–8 Mn atoms (when the interatomic distance d < 0.31 nm). The arrangements and interatomic distances of Mn atoms are important to the magnetism of the compound ErMn_5_Ge_3_, which will be discussed in Section 3.2 (magnetic properties). Figure 3 shows the environment of the nearest neighbor atoms of each Er or Mn atom in the structure of the compound ErMn_5_Ge_3_. The neighboring interatomic distances of ErMn_5_Ge_3_ (d < 0.31 nm) of each atom in the structure of ErMn_5_Ge_3_ are given in Table 3.

In the crystal structure of the new ErMn_5_Ge_3_ compound, individual MnGe_4_ tetrahedra are close in size, and the internal Mn atoms are positioned close to the MnGe_4_ tetrahedra centers. The MnGe_4_ tetrahedra and MnGe_5_ pyramids are closely aligned layer by layer along the [010] direction. All these are similar to other related structures of the RE-Mn-Ge ternary compounds. These compounds, such as the crystal structures of REMn_2_Ge_2_ and REMnGe, also contain MnGe_4_ tetrahedra, and are based on the MnGe_4_ tetrahedral coordination [2,3,4].

### 3.2. Magnetic Properties

**Figure 4:**
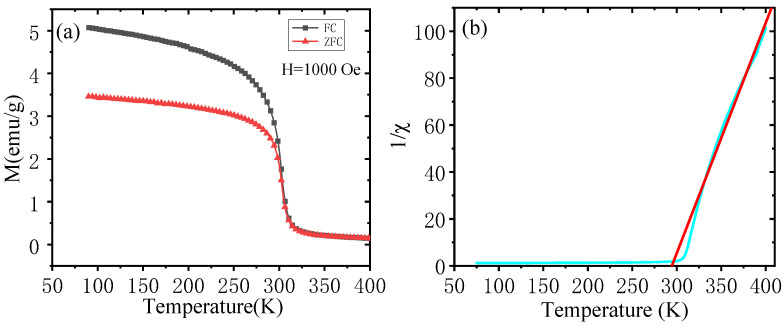
(**a**) Thermal-magnetic curve of ErMn_5_Ge_3_ (H = 1000 Oe, from 90 to 400 K). (**b**) The relationship between the reciprocal of magnetic susceptibility and temperature (blue line), the red line represents the fitting result of the Curie–Weiss law.

The thermomagnetic curves of FC and ZFC processes for the new compound ErMn_5_Ge_3_ were measured in the temperature range of 90 to 400 K under an external magnetic field of 1000 Oe, as shown in Figure 4a. The shapes of the magnetization curves are basically the same in FC and ZFC processes, and both of them show a gradual decrease in the magnetization at a defined temperature. The Curie temperature (*T_C_*) represents the transition point from the magnetically ordered state to the non-magnetically ordered state. The transition temperature *T_C_* is taken as the maximum value of the absolute value of the first-order derivative of the curve (|dM/dT|), which gives *T_C_* = 304 K for the new compound ErMn_5_Ge_3_. In the low-temperature range, the magnetization of ErMn_5_Ge_3_ is high, and decreases rapidly around *T_C_*, which suggests a certain magnetic ordering structure at low temperatures. It also can be seen in Figure 4a that the FC curve goes together with the ZFC curve down to around 300 K, and then the ZFC and FC curves are separated below 300 K for the new compound ErMn_5_Ge_3_, showing a large thermal hysteresis between the ZFC and FC curves. From Figure 4b, one can clearly see that ErMn_5_Ge_3_ is paramagnetic and obeys the Curie–Weiss law in the high temperature range. The reciprocal of the magnetic permeability (1/χ) and the temperature (*T*) basically satisfy a linear relationship above the Curie temperature for ErMn_5_Ge_3_. As described by the Curie–Weiss law

(1) $$\chi =\frac{C}{T-T_{C}}$$

where *χ* is the measured magnetic susceptibility of the new compound ErMn_5_Ge_3_, *C* represents the Curie constant (independent of temperature, related to the effective moment of the substance).

The effective magnetic moment can be further derived from the ion paramagnetism equation after obtaining the Curie constant:

(2) $$C=\frac{N_{A}\mu_{B}^{2}}{3k_{B}}\mu_{eff}^{2}$$

where *N_A_* is Avogadro’s constant, *N*_A_ = 6.023 × 10^23^ mol^−1^; *μ_B_* is Borel’s constant, *μ_B_* = 9.274 × 10^−24^ J/T; *k_B_* is Boltzmann’s constant, *k_B_* = 1.380649 × 10^−23^ J/K; and *μ_eff_* is the effective Bohr magneton number.

The Curie–Weiss law fitting results show that the Curie–Weiss temperature of the new compound ErMn_5_Ge_3_ is 297 K and the Curie constant is *C* = 1.019. The effective magnetic moment for the compound ErMn_5_Ge_3_ is obtained to be *μ_eff_* = p_eff_ *μ_B_* = 2.8 *μ_B_*.

**Figure 5:**
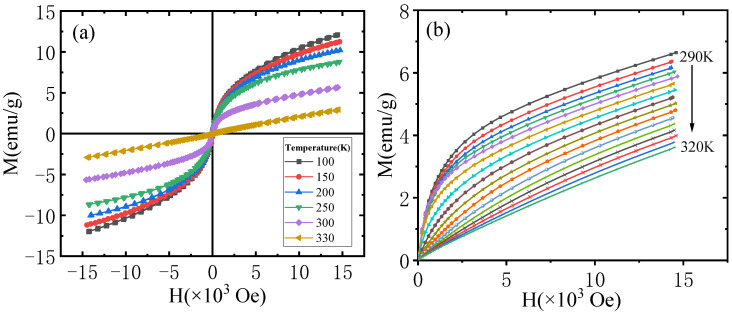
(**a**) The magnetization curve and hysteresis loop of ErMn_5_Ge_3_ (100 to 330 K). (**b**) Isothermal magnetization curves of ErMn_5_Ge_3_ around Curie temperature (from 290 to 320 K, each line is 2 K apart).

Figure 5a shows the magnetization curves of ErMn_5_Ge_3_ from 100 to 330 K with a maximum external magnetic field of up to 15,000 Oe. When the external field is low, the magnetization increases almost linearly with the increase in the external field, indicating the presence of antiferromagnetism. The magnetization of the compound ErMn_5_Ge_3_ increases with an increasing external field, while its growth rate decreases gradually. When the external field reaches the maximum of 15,000 Oe, the magnetization is approximately 12 emu/g at 100 K. It is clear that the magnetization continues to increase as the external field increases, and no magnetization saturation was observed.

Comparing the change in magnetization of the compound ErMn_5_Ge_3_ at different temperatures, the magnetization decreases gradually with increasing temperature, indicating the influence of thermal perturbation. The isothermal magnetization curve of ErMn_5_Ge_3_ from 100 to 300 K is a circular arc with a decreasing slope, which becomes a straight line with a constant slope at 330 K. At 330 K, the compound ErMn_5_Ge_3_ loses its magnetic order and exhibits paramagnetism. This process indicates that the Curie temperature of the compound ErMn_5_Ge_3_ is in the range of 300–330 K, which is consistent with the results of the above magnetization–temperature curves.

To further investigate the magnetic behavior of the samples near the Curie temperature, the isothermal magnetization (M-H) curves of the compound ErMn_5_Ge_3_ were measured every 2 K between 290 and 320 K. The results are shown in Figure 5b. It is obvious that the initial slope of the M-H curves of the compound ErMn_5_Ge_3_ decrease gradually as the temperature increases. Their arc gradually flattens out, eventually becoming a straight line. The maximum magnetization gradually decreases from 6.7 to 3.6 emu/g when the temperature rises from 290 to 320 K and shows a significant decrease in magnetic properties.

Isothermal magnetic entropy change in materials (Δ*S_M_*) can reflect the energy changes in magnetic objects in a magnetic ordered state, which is a type of energy change caused by changes in the magnetic field (*M*) inside the magnetic material and related temperature (*T*) changes. The maximum value of magnetic entropy change generally occurs near the phase transition temperature. It can be obtained from the Maxwell relation [27]:

(3) $$\Delta S_{M}\left(T,H_{max}\right)=S_{M}\left(T,H_{max}\right)-S_{M}\left(T,0\right)=\int_{0}^{H_{max}}\left(\frac{\partial M}{\partial T}\right)dH$$

Here, *S*, *M*, *H*, and *T* denote entropy, magnetization, applied field, and temperature. We used a simplified form of the formula:

(4) $$\Delta S_{M}=\sum_{i}\frac{M_{i+1}-M_{i}}{T_{i+1}-T_{i}}\Delta H$$

**Figure 6:**
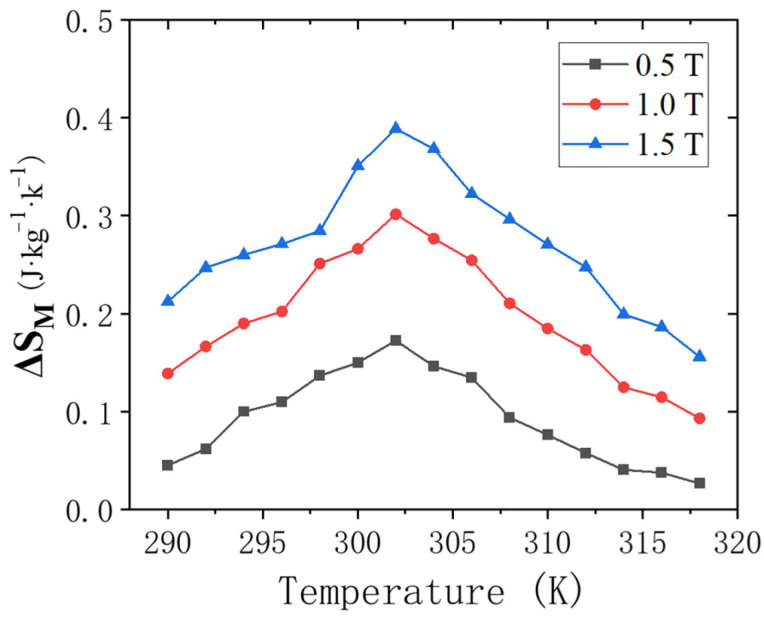
Temperature dependence of the magnetic entropy change at different amplitudes of change in the applied magnetic field 0.5 T, 1 T, and 1.5 T for ErMn_5_Ge_3_.

The maximum magnetic entropy change Δ*S_M_* of the new compound ErMn_5_Ge_3_ as a function of temperature was calculated for 0.5 T, 1 T, and 1.5 T magnetic fields near the Curie temperature (290–320 K), as shown in Figure 6. It can be seen that Δ*S_M_* reaches its maximum value at 302 K, which basically corresponds to the Curie temperature *T_C_* = 304 K of the new compound ErMn_5_Ge_3_, and the maximum magnetic entropy change Δ*S_M_* keeps increasing with the increase in the external field.

The magnetic properties of ErMn_5_Ge_3_ are relatively simple from the results of the magnetic measurements. The magnetization–temperature curve of ErMn_5_Ge_3_ is relatively smooth without any obvious fluctuation before rising from the low temperature (90 K) to the Curie temperature (*T_C_* = 304 K), indicating that ErMn_5_Ge_3_ remains as the same magnetic structure in the low temperature range (from 90 to about 300 K, the possibility of magnetic transformation at lower temperatures cannot be ruled out). No evidence of any magnetic phase transition or other structural changes was observed. Upon reaching the Curie temperature, the compound ErMn_5_Ge_3_ rapidly loses its magnetically ordered structure and turns to a paramagnetic state.

The interplay of magnetism with certain crystal symmetry leads to magnetic frustration or magnetic inhomogeneity/disorder [28]. A typical example, when the Mn atoms are arranged in a square triangle or a regular tetrahedral form in a Mn-based magnetic material (e.g., β-Mn structures), each Mn atom is adjacent to multiple equivalent Mn atoms at the same time, making simultaneous antiferromagnetic coupling impossible [29,30,31,32,33,34,35]. At this point, the Mn atoms have random coupling modes and magnetic moment directions, and they are even able to realize spin-glass states in some cases [29,30,31]. As shown in Figure 2b,c, the arrangement of Mn atoms in ErMn_5_Ge_3_ is extremely complex, as there are a large number of triangles and pyramid structures. Despite not being a typical tetrahedral structure, the complex Mn-Mn coupling makes the Mn atoms have a variety of magnetic moment choices. Different coupling modes present in a material at the same time, making for very low magnetic ordering. The magnetic moment of each Mn atom is more inclined to be consistent with the applied external field, and the magnetic ordering is obviously improved and result in a higher magnetization. This explains the apparent differences that emerge between the ZFC and FC processes.

On the other hand, the magnetic properties of ErMn_5_Ge_3_ are directly determined by its large number of Mn-Mn interactions. A large number of studies have shown that the Mn-Mn magnetic interactions are closely related to their distances in Mn-rich systems. In most cases, when the Mn-Mn distance is longer than a certain value, it supports ferromagnetism, while smaller distances contribute to antiferromagnetic ordering. This distance varies slightly with crystal structure and temperature and is usually considered to be about 0.285–0.287 nm at room temperature [11,12,13,14]. We use 0.286 nm here as a criterion for judging the form of magnetic coupling between Mn atoms.

We have calculated and listed the distances between various Mn atoms in the structure of ErMn_5_Ge_3_ in Table 3. It can be seen that the Mn-Mn distances in ErMn_5_Ge_3_ are relatively short, which would make many direct magnetic interactions possible. There are slightly differences in the different Mn-Mn distances, but they are all close to the critical distance of 0.286 nm, implying that there may exist both ferromagnetic interactions and antiferromagnetic interactions between magnetic moments in the structure of ErMn_5_Ge_3_. The coexistence and competition of a large number of ferromagnetic and antiferromagnetic Mn-Mn interactions further exacerbate the uncertainty of the Mn atomic magnetic moment. The magnetic behavior of ErMn_5_Ge_3_ is characterized by both ferromagnetism and antiferromagnetism in the low to room temperature range. For example, the Curie–Weiss fitting results of ErMn_5_Ge_3_ are basically consistent with those of ferromagnetic materials, while the magnetization curves show that ErMn_5_Ge_3_ have not reached their saturation magnetization reflecting certain antiferromagnetic features.

The Mn-Mn spacing can be adjusted by methods such as doping and applying external stresses, which affects the magnetic properties of ErMn_5_Ge_3_. It should also be noted that the Mn and Ge atoms in ErMn_5_Ge_3_ are relatively close to each other, and a large number of Mn-Ge interactions can also significantly affect the magnetic properties of the Mn atoms.

### 3.3. Electronic Structure

A computational model was constructed based on the crystal structure of ErMn_5_Ge_3_ obtained from the above XRD analysis (Section A), which is consistent with the size of the crystal protocell and contains 18 atoms. The dimensional parameters of the computational model used are α = β = γ = 90°, a = 3.8854 Å, b = 11.4027 Å, and c = 13.0525 Å. The geometrical optimization of the structural model was carried out to obtain the optimal structure, and the positions of the atoms before and after the optimization basically remained unchanged, which indicates that the structure can satisfy the computational conditions.

**Figure 7:**
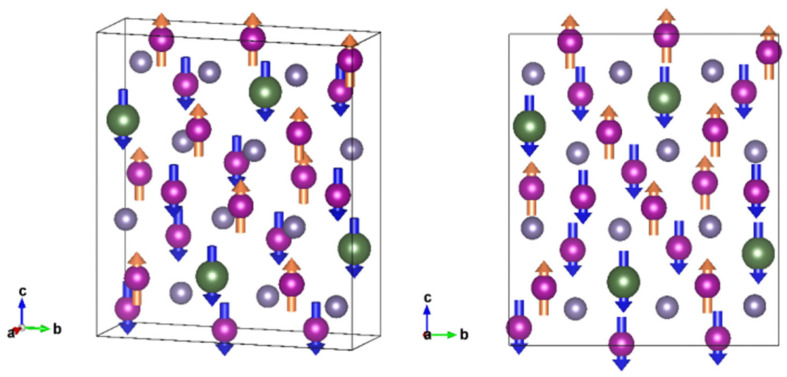
Setting of the magnetic coupling forms in the model (arrows only indicate direction, not size).

Preliminary calculations indicate that the magnetism of ErMn_5_Ge_3_ is mainly provided by Mn atoms, and no significant magnetism was observed in Er and Ge atoms. The setting of the initial magnetic moment will greatly affect the calculation results. After trying various configurations, it was found that using the combination of ferromagnetism and antiferromagnetism composed of Mn atoms can achieve more ideal calculation results. In the initial settings of this calculation, the distance between Mn-Mn atoms in the structure of ErMn_5_Ge_3_ determines whether their coupling is ferromagnetic or antiferromagnetic (the critical distance is set to be 2.86 Å). When the distance is greater than the critical distance, the two atoms are ferromagnetic coupling(magnetic moment symbol is the same). Conversely, when the distance is less, the two atoms are antiferromagnetically coupled (magnetic moment symbol is opposite). The initial setting of the magnetic moments in this calculation is consistent with the results of the previous discussion on magnetism, where the Mn atoms determined the magnetic properties of the structure. The form of magnetic coupling is determined by the Mn atomic spacing. Based on the results of the previous magnetic optimization, the initial magnetic moment of Mn atoms is set to 3 μ_B_. Manually set the magnetic coupling mode for each Mn atom. Due to the small influence of Er and Ge atoms on the magnetic properties of ErMn_5_Ge_3_, the initial magnetic moments are set to 1 μ_B_ and 0, respectively. The initial magnetic moments of the rare-earth atoms are set to be extremely small, which are both reference to actual findings in the same structure and in agreement with our previous calculations. Large initial magnetic moments have been tried toassign to the rare-earth atoms; however; their moments were all optimized to be very small after magnetic calculations. During the calculation, several initial magnetic moments are set for Mn, Er, and Ge atoms, and the final magnetic results show that the Mn atom has a larger magnetic moment while the other two atoms (Er and Ge) have almost no magnetic moment. The setting of magnetic coupling forms for each atom is shown in Figure 7. Notice in the figure that the directions do not represent the actual magnetic moment directions, but rather indicate the form of interatomic magnetic coupling. The same direction represents ferromagnetic coupling, and the opposite direction indicates antiferromagnetic coupling.

After magnetic calculation, the mode of magnetic coupling between individual atoms is consistent with the beginning. The magnetic moment of Mn atoms is optimized to 4.4~4.6 μ_B_ after calculation, while Er and Ge atoms have very small magnetic moments of about 0.12 μ_B_ and 0.02 μ_B_, respectively. At this point, the magnetism of the compound is mainly determined by Mn atoms, and the total magnetic moment of the model is 8.74 μ_B_, and the free energy of the system is −206.42 eV. The magnetic moment of all Mn atoms for ErMn_5_Ge_3_ in the calculation results is about 4.5 μ_B_, slightly larger than the 3 μ_B_ obtained from neutron diffraction testing in the experiment at similar Mn-Mn interatomic distances [19]. Considering that first-principles calculations yield the optimal structure under ideal conditions, it is normal for the calculated atomic magnetic moment to be slightly greater than the experimental results.

**Figure 8:**
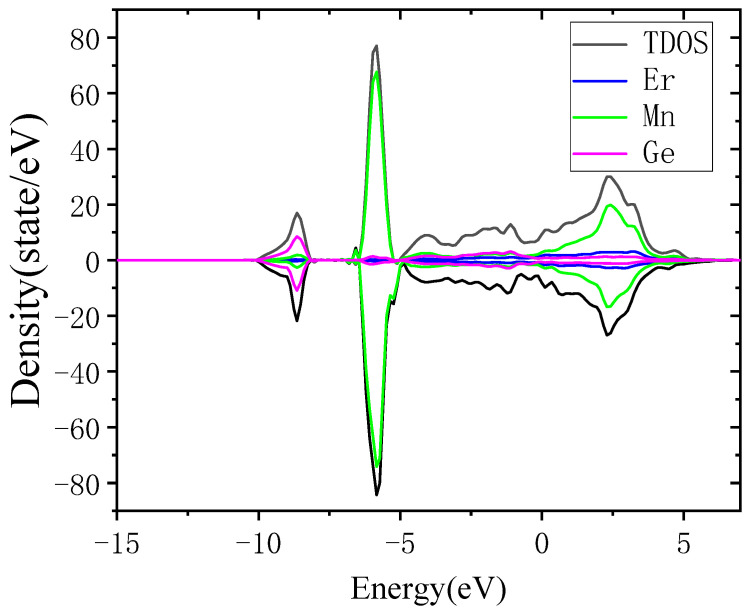
Total density of states and contribution of different elements (positive density represents spin up and negative density indicates spin down).

The calculation results of the electron density of states (total density of states, TDOS) for ErMn_5_Ge_3_ are shown in Figure 8. The number of electrons at the Fermi level is not zero, indicating that the new compound ErMn_5_Ge_3_ has obvious metallic characteristics. The electron density of states in the spin up and spin down directions is basically symmetrical. Although a single Mn atom has a large magnetic moment, the magnetic property of the entire material of ErMn_5_Ge_3_ is weak and in an antiferromagnetic state. A wide energy distribution of the electron density of states for ErMn_5_Ge_3_ can be observed near the Fermi surface, and it can be seen that the energy of the bonded electrons in the structure of ErMn_5_Ge_3_ is mainly between −5 eV and 6 eV.

**Figure 9:**
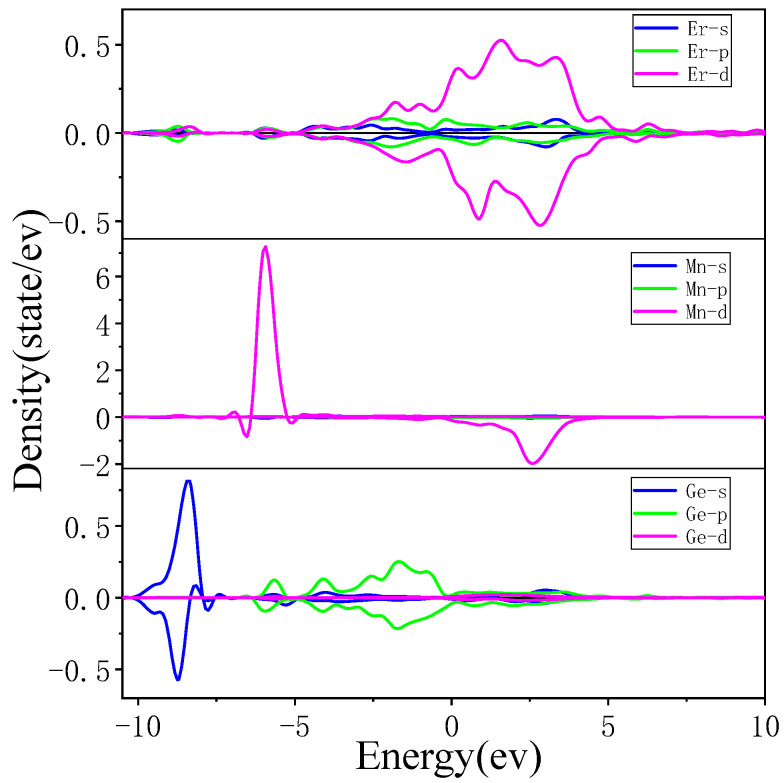
The PDOS of Er, Mn, and Ge monatomic (positive density indicates spin up and negative density represents spin down).

After analyzing the density of the states of individual atoms of ErMn_5_Ge_3_, it was found that although the atoms of ErMn_5_Ge_3_ are in different environments, the distribution of the electronic density of states for the same element is basically the same, and the values of their magnetic moments are also basically the same. The partial wave density of the states of the individual atoms of different elements for ErMn_5_Ge_3_ is shown in Figure 9.

The density of the states of the Er and Mn atoms of ErMn_5_Ge_3_ is mainly determined by the d-state electrons, while the density of the states of the Ge atoms is provided by the s-state in the range of −10 to −8 eV, and by the p-state in the higher energy range. Combined with the TDOS diagram, the feature at the energy from −10 to −8 eV mainly reflects the Ge-s states and large sharp peaks from −6.5 to −5 eV mainly reflect the Mn-d states. The magnetism of ErMn_5_Ge_3_ mainly comes from Mn atoms, provided by their asymmetric 3d orbitals at about −6 eV.

The magnitude of the energy width of the wave function can reflect the strength of electron localization and the ability of electrons to participate in bonding: small band width, strong electron localization, weak atomic orbital expansion, and a small ability of electrons to participate in bonding. On the contrary, the degree of electron localization is weak, the atomic orbital expansion is strong, and the ability of electrons to participate in bonding is greater. Figure 9 is intended to show energy levels and analyze bonding relationships. Positive density indicates spin up and negative density represents spin down. Observing the density of the states of each element (Figure 9), the atomic density distribution of the Er and Ge of ErMn_5_Ge_3_ is relatively wide, mainly playing a bonding role, bonding in the range of −5 to 0 eV, forming the matrix of ErMn_5_Ge_3_. The outer electrons of the Mn atoms of ErMn_5_Ge_3_ not only bond with Er atoms in the range of 0–5 eV but also have strong electron localization around −5 eV, forming a relatively high peak.

The calculation results further indicate that ErMn_5_Ge_3_ is a framework composed of Er Ge, filled with Mn atoms and providing a magnetic structure, as mentioned earlier. Although the surrounding environment of each Mn atom is different, a large number of Mn-Mn and Mn-Ge bonds make the density of the states of each atom tend to be uniform. During this process, Ge atoms not only form the framework of ErMn_5_Ge_3_ but also serve as intermediaries for electronic interactions, enabling long-distance interactions between Mn-Mn and Mn-Er at longer distances.

From the calculation results, it can be seen that the structure of ErMn_5_Ge_3_ is stable under this model, and its magnetism conforms to the experimental results. It is believed that this structure can well reflect the actual situation. During the calculation process, we noted that the free energy difference between different magnetic configurations is relatively small. Changing the direction of the magnetic moment of one or more Mn atoms in the structure of ErMn_5_Ge_3_ will not cause a significant impact on the system energy but will significantly affect the overall magnetic performance. It is believed that due to the complex interactions between multiple Mn atoms in the surrounding environment, the direction of their magnetic moments may easily change. A large number of Mn atoms in the ErMn_5_Ge_3_ compound coexist and compete with each other in ferromagnetic and antiferromagnetic interactions, resulting in its unique magnetic properties. This is also correspond with the results of previous magnetic tests.

## 4. Conclusions

In summary, the novel compound ErMn_5_Ge_3_ was successfully synthesized, and its crystal structure was determined, and the magnetic properties in the range of 90–400 K of ErMn_5_Ge_3_ were investigated by experimental methods and the first principle calculation. The novel compound ErMn_5_Ge_3_ crystallizes in the YNi_5_Si_3_-type orthorhombic structure with the space group Pnma (No. 62).

Magnetic measurement results show that ErMn_5_Ge_3_ has a Curie temperature of 304 K and the effective magnetic moment for the compound is *μ_eff_* = p_eff_ *μ_B_* = 2.8 *μ_B_*. The difference between the ZFC and FC curves at the lower temperature region shows the unique magnetic structure of ErMn_5_Ge_3_. Due to the uncertainty in the magnetic moments of Mn atoms, the magnetic ordering of ErMn_5_Ge_3_ is low and a magnetic frustration-like phenomenon exists. This suggeststhe coexistence and competition between ferromagnetism and ferromagnetism in ErMn_5_Ge_3_, which related to its numerous and relatively short Mn-Mn atomic spacing. The isothermal magnetization curves of the compound ErMn_5_Ge_3_ were measured, and the maximum magnetic entropy change Δ*S_M_* of ErMn_5_Ge_3_ was also obtained.

First-principles calculations confirm that the magnetic properties of ErMn_5_Ge_3_ are mainly provided by the Mn atoms, and its magnetic moment is calculated to be about 4.5 *μ_B_*. A possible magnetic structure model of simultaneous ferromagnetic–antiferromagnetic coupling between Mn atoms based on the Mn atom spacing, which can well explain the magnetic performance of ErMn_5_Ge_3_.

## Figures and Tables

**Table 1:** Crystallographic data and Rietveld refinement results for ErMn_5_Ge_3_.

Space Group	Pnma		
Density (g/cm^3^)	7.578		
Cell parameters	a = 13.0524(6) Å	b = 3.8853(7) Å	c = 11.4027(4) Å
Cell volume	v = 578.274 Å^3^		
Z	2		
Radiation	Cu Kα		
2θ(°)	10–110		
Refinement results	R_p_ = 2.687%	R_wp_ = 3.649%	R_exp_ = 1.689%
	χ^2^ = 4.66		

**Table 2:** Atomic and thermal parameters for ErMn_5_Ge_3_.

Atom	Wyckoff Sites	Atomic Parameters			
		x	y	z	Uiso (Å^2^)
Er	4(c)	0.2041(3)	1/4	0.9243(4)	0.04893
Mn1	4(c)	0.4765(8)	1/4	0.7738(6)	0.02658
Mn2	4(c)	0.9948(9)	1/4	0.0832(7)	0.03717
Mn3	4(c)	0.6929(6)	1/4	0.2643(8)	0.02495
Mn4	4(c)	0.1858(3)	1/4	0.6293(4)	0.02956
Mn5	4(c)	0.4423(2)	1/4	0.0891(6)	0.02773
Ge1	4(c)	0.6273(1)	1/4	0.0365(6)	0.07505
Ge2	4(c)	0.3820(6)	1/4	0.2457(2)	0.04569
Ge3	4(c)	0.8773(4)	1/4	0.9139(9)	0.03684

**Table 3:** Interatomic distances of ErMn_5_Ge_3_ (d < 0.31 nm).

Atom	Atom	D (nm)	Atom	Atom	d (nm)	Atom	Atom	d (nm)
Er–	2 Ge1	0.293910(8)	Mn3–	1 Ge1	0.217398(9)	Ge1–	2 Er	0.293910(8)
	2 Ge2	0.303122(8)		1 Ge2	0.247105(10)		2 Mn1	0.283763(7)
	2 Ge3	0.288146(7)		2 Ge3	0.274341(7)		1 Mn3	0.217398(9)
Mn1–	2 Ge1	0.283763(7)		2 Mn1	0.297679(8)		1 Mn4	0.260620(11)
	2 Ge2	0.268845(7)		2 Mn_4_	0.278294(6)		1 Mn5	0.257985(6)
	1 Ge3	0.250330(9)	Mn4–	1 Ge1	0.260620(11)		2 Mn5	0.248786(9)
	2 Mn2	0.293855(9)		2 Ge2	0.251405(6)	Ge2–	2 Er	0.303122(8)
	2 Mn3	0.297679(8)		1 Ge3	0.254796(10)		2 Mn1	0.268845(7)
	1 Mn4	0.294592(10)		1 Mn1	0.294592(10)		1 Mn2	0.246585(8)
	1 Mn5	0.302866(13)		2 Mn3	0.278294(6)		1 Mn3	0.247105(10)
	2 Mn5	0.309311(8)		2 Mn5	0.277349(6)		2 Mn4	0.251405(6)
Mn2–	1 Ge2	0.244353(8)	Mn5–	1 Ge1	0.257985(6)	Ge3–	2 Er	0.288146(7)
	1 Ge3	0.246585(8)		2 Ge1	0.248786(9)		1 Mn1	0.250330(9)
	2 Ge3	0.256044(7)		1 Ge2	0.2.51134(10)		1 Mn2	0.246585(8)
	2 Mn1	0.293855(9)		1 Mn1	0.3.02866(13)		2 Mn2	0.256044(7)
	2 Mn2	0.272008(8)		2 Mn1	0.3.09311(8)		2 Mn3	0.274341(7)
				2 Mn4	0.2.77349(6)		1 Mn4	0.254796(10)
				2 Mn5	0.2.59531(6)			

## Data Availability

The original contributions presented in this study are included in the article. Further inquiries can be directed to the corresponding authors.
